# Influence of glycoprotein IIb/IIIa inhibitors on bleeding events after successful resuscitation and percutaneous coronary intervention

**DOI:** 10.1007/s00392-019-01518-7

**Published:** 2019-07-12

**Authors:** Paul Marc Biever, Dawid Leander Staudacher, Jonas Degott, Corinna Nadine Lang, Christoph Bode, Tobias Wengenmayer

**Affiliations:** 1 Department of Cardiology and Angiology I, Faculty of Medicine, Heart Center Freiburg University, University of Freiburg, Hugstetter Strasse 55, 79106 Freiburg, Germany; 2 Department of Medicine III (Interdisciplinary Medical Intensive Care), Medical Center, Faculty of Medicine, University of Freiburg, Hugstetter Strasse 55, 79106 Freiburg, Germany

**Keywords:** Glycoprotein IIb/IIIa antagonists, Glycoprotein IIb/IIIa inhibitors, Anticoagulation, Bleeding, Cardiac arrest, Resuscitation

## Abstract

**Aim:**

Cardiac arrest is the most serious complication in acute coronary syndromes. Glycoprotein IIb/IIIa inhibitors (GPI) are used in selected acute coronary syndrome patients. If the use of GPI leads to an increase in bleeding events and influences survival in patients after cardiac arrest is unknown.

**Methods:**

We report retrospective data of a single center registry of patients after successful intra- and out-of-hospital cardiac arrest between 2002 and 2013. Inclusion criteria were survival for at least 6 h and successful percutaneous coronary intervention (PCI) within the first 24 h. Patients treated with other fibrinolytic agents or being supported by an extracorporeal life support system were excluded from the analysis.

**Results:**

310 patients were included in our study. 204 received GPI (GPI+), 106 did not (GPI−). Patients in the GPI+ group were significantly younger (62.8 vs. 68.0 years, *p* < 0.001) and had larger myocardial infarction sizes (maximum creatine kinase 3407 vs. 1450 U/l, *p* < 0.001). CPR duration, SOFA score and first lactate did not differ between the groups. Any bleeding occurred significantly more often in the GPI+ group (83.3% vs. 67.0%, *p* = 0.001). Decline of hemoglobin within the first 24 h was higher in the GPI+ group (−1.59 ± 1.71 mg/dl vs. −0.88 ± 1.95 mg/dl, *p* = 0.004), number of transfused packed red blood cells in the first 4 days, however, were similar (1.18 ± 0.40 vs. 0.90 ± 0.41 packs, *p* = 0.378). Survival at ICU discharge was significantly higher in the GPI+ group (77.5% vs. 63.2%, *p* = 0.008). The use of GPI was an independent predictor of hospital survival (OR 3.07, CI 1.31−7.20, *p* = 0.010). The positive effect for GPI persisted after nearest neighbor propensity score matching including 144 patients (OR 3.27, 95% CI 1.48−7.21, *p* = 0.003).

**Conclusion:**

After cardiac arrest, bleeding incidence was significantly higher in patients treated with GPI. Incidence of bleedings requiring transfusion, however, was similar. In this retrospective analysis, the use of GPI was an independent predictor of hospital survival. We suggest that GPI may not be withheld from cardiac arrest survivors due to potential risk of bleeding.

**Graphic abstract:**

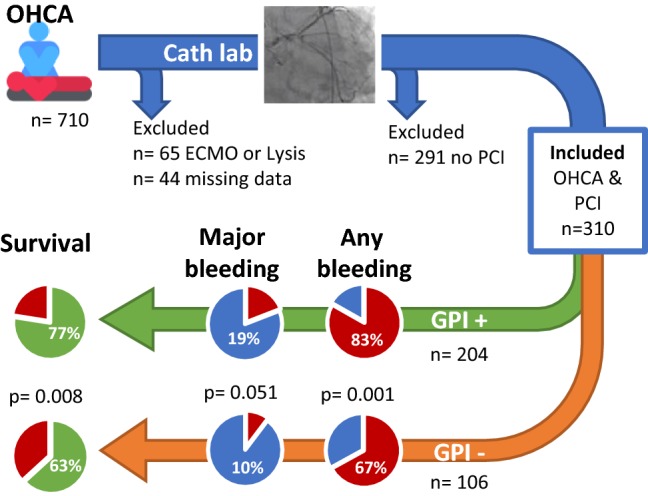

## Introduction

Sudden cardiac death, or out-of-hospital cardiac arrest (OHCA), is one of the leading causes of death in western countries [1]. Tremendous efforts are undertaken to increase survival rates by optimizing the “chain of survival”, which includes post-resuscitation care and the identification of the underlying pathology. Clinically significant coronary disease is frequent in resuscitated patients. Therefore, early coronary angiography is recommended in patients with suspected myocardial infarction [2].

Antiplatelet therapy has to be immediately implemented after revascularization of the culprit lesion which might be difficult and delayed in an instable and intubated patient and an oral application formula of the medication. Therapeutic, mild hypothermia might foster a trend towards more bleeding [3]. Increased bleeding might be explained by reduced platelet adhesion in lower body temperature [4].

On the other side, in hypothermia might be an elevated risk for thrombotic complications. Hypothermia induces reduced metabolism in general, which might also implicate the metabolism of the platelet antagonists clopidogrel, ticagrelor, and prasugrel. Moreover, application and resorption of oral medications in critical ill patients are often delayed. A reduced effect of platelet inhibitors might explain the increased rates of stent thrombosis in resuscitated patients [5, 6]. Therefore, glycoprotein IIb/IIIa inhibitors (GPI) might be beneficial in patients after cardiac arrest.

GPI are intravenous platelet antagonists which used to be given widely during percutaneous coronary intervention (PCI), particularly, in cases of acute coronary syndromes Indications for the application of GPI were high-risk patients with a substantial thrombus burden, a high-risk anatomy, intra-procedural complications, e.g., dissections, perioperative bridging, or the missing possibility of oral administration of the P2Y12 receptor blockers (like ticagrelor, prasugrel, and clopidogrel). Indeed, the addition of GPI to the standard care with acetylsalicylic acid and heparin reduced the risk of ischemic complications significantly. On the other hand, the use of GPI increases the rates of bleeding complications, especially when combined with heparin [7–9]. Current guidelines are reserving use of GPI for bailout situations with high thrombotic burden [10].

All data on GPI, however, are derived from hemodynamically stable patients with acute coronary syndromes. The impact of the increased bleeding events in OHCA patients treated with GPI on the severity of bleeding, transfused blood products or survival is unknown. The aim of this study, therefore, was to investigate the influence of GPI on bleeding complications and survival in patients after cardiac arrest.

## Methods

We retrospectively analyzed medical records of patients who had been successfully resuscitated due to a suspected coronary cause and were treated at the University Heart Center Freiburg between 01/2002 and 06/2013. Inclusion criteria were survival for at least 6 h and a successful PCI within the first 24 h. Patients treated with a fibrinolytic agent or on extracorporeal life support system were excluded from the analysis. Analysis was blinded to patient identity and was covered by an ethics approval (Ethics Committee of Albert-Ludwigs University of Freiburg, file number 183/10).

### Data collection

Using a computerized search, we identified a total of 710 individual patients receiving a coronary angiography after resuscitation. By manual review inclusion criteria were verified. Data collection were based on a tabular listing of patient characteristics using (Microsoft Excel). For some items (like bleeding events), multiple selections were possible while most other items only single selection was possible (like gender). Calculated data included SOFA, Crusade and Acuity Scores.

### Anticoagulation und platelet inhibitors after PCI

Indication for application of platelet inhibitors and GPI after PCI in resuscitated patients was left to the treatment of the interventional cardiologist and was in accordance to guidelines at that time. GPI was dosed by body weight and kidney function along unfractionated heparin with a fixed dose of 500 U/h. Patients with myocardial infarction not receiving GPI obtained unfractionated heparin aiming at a partial thromboplastin time (PTT) of 50–70 s until creatine kinase resolved, according to a local standard.

### Post-resuscitation management on intensive care unit

Post-resuscitation care was performed following a standardized treatment protocol including targeted temperature management. Indication for therapeutic hypothermia was persistent comatose state after return of spontaneous circulation (ROSC). Standard cooling management was 33 °C for 24 h using Coolgard^®^, Emcools^®^, Arctic Sun^®^ or Thermo^®^ devices.

### Outcome variables

All outcome variables were evaluated by manual search of medical and patient records. Collected outcome variables were number of bleeding events, the drop in hemoglobin, the number of transfused units of packed red blood cells, rates of definite stent thrombosis and mortality at discharge from hospital.

Bleedings were categorized using the BARC [20] classification (brief: BARC0—no bleeding; BARC1—minimal bleeding; BARC2—bleeding that needs further diagnostic or therapeutic steps, BARC3—bleeding plus drop in hemoglobin; BARC4—CABG related bleeding, BARC5—fatal bleeding). Underreporting of minor bleedings (BARC1) cannot be excluded. A drop of hemoglobin > 3 mg/dl or any red blood cell transfusion was considered to be a major bleeding (BARC3). Bronchoscopic evidence of pulmonary bleeding, hemothorax, gastrointestinal, urethral, access site, nose, and pharyngeal bleeding were considered as bleedings that needed further diagnostic or therapeutic steps.

Trigger for transfusion of packed red blood cell was a hemoglobin value < 8 mg/dl.

### Statistical analysis

Relevant data were integrated in standardized tables. For data analysis, SPSS (version 23, IBM Statistics) and Prism (version 5, GraphPad) were employed. For statistical analysis, unpaired *t* test, Fisher’s exact test, *Z*-score and Wald test were used as applicable and a *p* value of < 0.05 was considered statistically significant. Data are given as *n* (%), mean ± standard deviation or odds ratio (OR) with 95% confidence interval (CI) if not stated otherwise. Propensity score matching was performed using SPSS with a nearest neighbor matching algorithm (caliper 0.2) using the independent predictors of hospital survival (age, CPR duration and SOFA score) as detected by multivariate logistic regression analysis of the whole cohort.

## Results

**Fig. 1:**
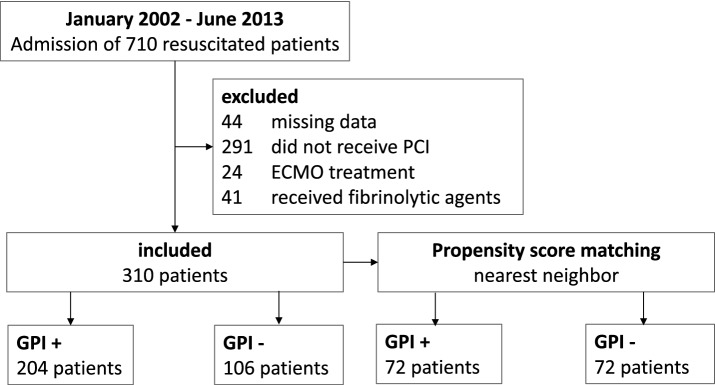
Flowchart indicating number of included and excluded patients. Data are given as number of patients

From January 2002 to June 2013, 710 patients were admitted to our hospital after OHCA. A total of 400 patients were excluded from analysis (44 for missing data, 24 for ECMO, 41 for fibrinolytic agent and 291 for unsuccessful or not performed PCI). Therefore, 310 datasets could be included (Fig. 1). 204 patients did receive GPI (GPI+) while 106 patients did not (GPI−). In 25 individuals receiving GPI the interventional cardiologist detected signs of high thrombotic burden or encountered a coronary no-reflow situation after stenting. In the other cases it was the individual decision of interventional cardiologist at that time. In average, patients in the GPI+ group were significantly younger (62.8 vs. 68 years, *p* = 0.001). The two groups did not differ in terms of distribution of sex, CPR duration, SOFA score, first available lactate or pH. In the GPI− group there were more patients on oral anticoagulation with phenprocoumon resulting in higher INR. Creatinine values were significantly higher in the GPI− group. Bleeding scores including Acuity and Crusade were significantly lower in the GPI+ group at initial presentation, as calculated retrospectively. Patients in the GPI+ group had a worse pulmonary function considering the Horowitz Index (GPI+ 233 vs. GPI− 333, *p* = 0.001) (Table 1).

**Table 1:** The characteristics of patients on admission

	GPI+	GPI−	*p* value
*n*	204	106	
Age (years)	62.85 ± 12.72	67.97 ± 13.07	**0.001**
Female sex (*n*)	40 (19.6%)	27 (25.5%)	0.234
CPR duration (min)	16.15 ± 1.88	13.61 ± 2.42	0.104
SOFA estimated mortality (%)	38.42 ± 2.32	39.93 ± 3.58	0.470
First lactate (mmol/l)	4.34 ± 0.59	5.15 ± 0.93	0.133
First pH	7.28 ± 0.02	7.27 ± 0.02	0.450
First creatinine	1.26 ± 0.97	1.75 ± 1.48	**0.001**
PEEP at 24 h (mbar)	6.90 ± 2.83	6.76 ± 2.77	0.723
Horowitz Index	233 ± 130	333 ± 140	**0.001**
Maximum creatine kinase (U/l)	3407 ± 5732	1450 ± 1803	**0.001**
Maximum troponin T (ng/ml)	4.62 ± 7.03	1.51 ± 2.76	**0.001**
Medication			
Oral anticoagulation	19 (9.3%)	20 (18.9%)	**0.016**
First INR	1.26 ± 0.09	2.01 ± 0.40	**0.001**
Scores			
Acuity score	23.29 ± 1.02	26.36 ± 1.63	**0.001**
Crusade score	44.65 ± 1.77	51.04 ± 2.72	**0.001**

*p* value reported in bold if difference significant (*p* < 0.05)

Data are given as mean ± standard deviation or number of patients (percent of all patients in group)

### Procedural and post-procedural characteristics

Table 2 shows treatment with acetylsalicylic acid, P2Y_12_ inhibitors and unfractionated heparin. Both groups had prolonged PTT values which was similar between the groups (ANOVA, *p* = 0.948). Focusing at day 2, PTT was significantly lower in den GPI+ group when compared to the GPI− group. Adherence to therapy with acetylsalicylic acid was high in both groups. The use of Clopidogrel was more frequent in the GPI+ group (85.5 vs. 68.9%, *p* = 0.001) whereas novel P2Y_12_ inhibitors were used more often in the GPI− group (11.3% vs. 23.6%, *p* = 0.004).Therapeutic mild hypothermia (33 °C) was induced in 71.9% of the cases. This therapy was more frequent in the GPI+ group than in the GPI− group (76.0% vs. 64.2%, *p* = 0.028) (Table 2).

**Table 2:** Information about treatment with platelet inhibitors, hypothermia, and activated partial thromboplastin time over the time

	GPI+	GPI−	*p* value
*n*	204	106	
Initial treatment			
Acetylsalicylic acid	196 (96.6%)	101 (95.3%)	0.584
Clopidogrel	175 (85.5%)	73 (68.9%)	**0.001**
Other P2Y_12_ inhibitors	23 (11.3%)	25 (23.6%)	**0.004**
Hypothermia	155 (76.0%)	68 (64.2%)	**0.028**
Lab-PTT			
PTT d1 (s)	90.13 ± 55.58	81.92 ± 53.31	0.215*
PTT d2 (s)	65.96 ± 38.69	85.54 ± 46.16	0.001*
PTT d3 (s)	72.92 ± 32.38	65.92 ± 27.31	0.093*
PTT d4 (s)	60.54 ± 24.15	59.95 ± 24.29	0.321*

*p* value reported in bold if difference significant (*p* < 0.05)

Data are given as mean ± standard deviation or number of patients (percent of all patients in group)

*p value for ANOVA analysis for PTT day 1–4: 0.948

### Bleedings

Any bleeding occurred in 83.3% of the GPI+ patients and in 67.0% of the GPI− cases (*p* = 0.001). In the GPI+ group, significant more bleedings at the access sites of central line and arterial sheath were recorded along bleedings in the gastrointestinal tract (Table 3). Incidence for hemothorax, pulmonary bleeding, epistaxis, or urethral bleeding was similar in both groups. We recorded three intracranial bleedings in the GPI− group, all of which were associated with a blunt trauma after collapse. In the GPI+ group, no intracranial bleeding occurred.

**Table 3:** Bleeding complication, their classifications and number of transfused blood products

	GPI+	GPI−	*p* value
*n*	204	106	
Survival	158 (77.5%)	67 (63.2%)	**0.008**
Bleeding events			
Any bleeding	170 (83.3%)	71 (67.0%)	**0.001**
Bleeding at central line, arterial sheath	125 (61.3%)	47 (44.3%)	**0.004**
Pulmonary bleeding (evidence by suctioning of endotracheal tube)	88 (43.1%)	35 (33%)	0.084
Epistaxis	32 (15.7%)	9 (8.5%)	0.076
GI bleeding	25 (12.3%)	5 (4.7%)	**0.033**
Urethral bleeding	15 (7.4)	6 (5.7%)	0.574
Pulmonary bleeding (evidence bronchoscopy)	5 (2.5%)	6 (5.7)	0.147
Hematothorax	2 (1%)	1 (1%)	0.975
Cerebral bleeding	0 (0%)	3 (2.8%)	**0.016**
Lab			
Hb d1 (mg/dl)	13.36 ± 0.28	12.52 ± 0.52	**0.004**
Hb d2 (mg/dl)	11.68 ± 0.30	11.67 ± 0.46	0.972
Hb d3 (mg/dl)	10.88 ± 0.33	11.37 ± 0.45	0.088
Hb d4 (mg/dl)	10.28 ± 0.24	10.68 ± 0.48	0.108
Drop of Hb d1 vs. d2	− 1.59 ± 1.71	− 0.88 ± 1.95	**0.002**
Drop of Hb d1 vs. d3	− 2.41 ± 2.23	− 1.25 ± 2.25	< 0.001
Drop of Hb d1 vs. d4	− 3.12 ± 1.,67	− 2.08 ± 2.66	< 0.001
Drop of hemoglobin > 3 mg/dl d1 vs. d4	39 (19.1%)	11 (10.3%)	0.051
Blood products			
Packed red cells d1-4 (*n*)	1.18 ± 0.40	0.90 ± 0.41	0.378
Packed red cells total (*n*)	1.62 ± 0.47	1.34 ± 0.62	0.480
Platelet concentrate d1-4 (*n*)	0.07 ± 0.09	0.11 ± 0.17	0.651
Platelet concentrate total (*n*)	0.09 ± 0.11	0.13 ± 0.18	0.692
Fresh frozen plasma d1-4 (*n*)	0.42 ± 0.28	0.29 ± 0.22	0.562
Fresh frozen plasma total (*n*)	0.45 ± 0.29	0.35 ± 0.23	0.646
BARC criteria			
BARC 0	34 (16.7%)	35 (33.0%)	< 0.001
BARC 1	79 (38.7%)	40 (37.7%)	0.012
BARC 2	56 (27.5%)	24 (22.6%)	< 0.001
BARC 3	36 (17.6%)	6 (5.7%)	< 0.001
BARC 5	0 (0%)	3 (2.8%)	< 0.001
Stent thrombosis	9 (4.4%)	1 (0.9%)	0.101

*p* value reported in bold if difference significant (*p* < 0.05)

Data are given as mean ± standard deviation or number of patients (percent of all patients in group)

On arrival in the Cath lab average hemoglobin (Hb) was higher in the GPI+ group (13.4 vs. 12.5 g/dl, *p* = 0.004). Hb declined over the first days after cardiac arrest and was not significant different between the both groups (Table 3). When evaluating the difference between initial Hb and the Hb 3 days after cardiac arrest (the drop of Hb), we found a significantly higher drop in the GPI+ group (−3.1 vs. −2.1, *p* < 0.001). A drop in Hb > 3 mg/dl at day 1, corresponding to a BARC3 bleeding, occurred in 19.1% of patients in the GPI+ group and in 10.3% in the GPI− group (*p* = 0.051). The amount of transfused packed red blood cells did not differ between the groups.

### Size of myocardial infarction and stent thrombosis

Maximum creatinine kinase (CK) and troponin T were significant higher in the GPI+ group (3407 vs. 1450U/l, *p* = 0.001 and 4.6 vs. 1.5 ng/ml, *p* = 0.001, respectively) (Table 1). Rates of acute stent thrombosis were 3.2% in the whole cohort. Incidence was higher in the GPI+ group, but did not reach statistical significance (GPI+ : 4.4% vs. GPI−: 0.9%; *p* = 0.101) (Table 3).

### Survival and predictors of survival

**Fig. 2:**
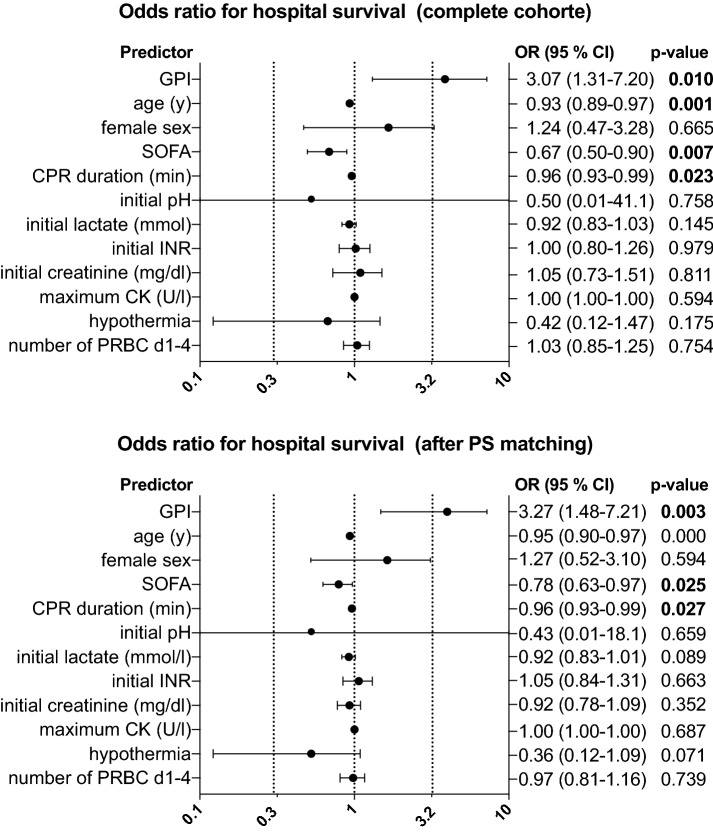
Graphs showing multivariate logistic regression analysis with odds ratio (95% confidence interval) of different predictors for hospital survival in patients after resuscitation and percutaneous coronary intervention (PCI). Upper figure shows predictors for the whole cohort, whereas lower figure refers to the matched cohort

Hospital survival was significantly better in the GPI+ group than in the GPI− group (77.5% vs. 63.2%, *p* = 0.008). We performed a multivariate logistic regression analysis to identify independent predictors of hospital survival. Therapy with GPI, age, CPR duration, and SOFA score turned out to be independent predictors of hospital survival (Fig. 2). Odds ratio for the use of GPI regarding hospital survival was 3.07 (95% CI 1.31–7.20, *p* = 0.010). These independent predictors of hospital survival (excluding GPI therapy) were incorporated in a propensity score matching algorithm (nearest neighbor) to adjust for bias. After propensity score matching, GPI therapy remained a significant, independent predictor of hospital survival (Fig. 2, *p* = 0.003).

## Discussion

Our data suggests that application of glycoprotein IIa/IIIb inhibitors after PCI in cardiac arrest patients is associated with improved hospital survival rates.

This finding is remarkable since we also found significantly more bleeding in the GPI group. And for patients with acute coronary syndromes, higher bleeding rates are linked to higher mortality [11]. This paradox might be explained by the fact that this research focuses on patients after cardiac arrest, only.

Patients after cardiac arrest and acute coronary syndrome may have a complex coronary artery disease with high thrombotic burden or coronary dissection which can be described as a prothrombotic state. Moreover, therapeutic hypothermia—which is installed in the majority of resuscitated patients—itself may increase the risk of stent thrombosis [5]. The reduced efficacy of antiplatelet drugs like P2Y12 inhibitors during hypothermia is a possible explanation [12].

Bleeding complications were more frequent in the GPI+ group, however, with a doubtful clinical implication: most of the bleeding complications were minor bleedings (BARC 1 and 2) in terms of catheter access site bleedings or bloody secretion after endotracheal suctioning. Furthermore, rate of transfusions was similar in both groups indicating that major bleeding was similar in both groups.

A possible explanation of our finding of better hospital survival in the GPI+ group is a bias towards sicker patients being allocated to the GPI− group. Indeed, GPI+ patients were younger and had better renal function. Interestingly, when performing a multivariate logistic regression analysis, first creatinine value was no significant predictor of survival. Other known predictors of survival including SOFA score, initial lactate or pH, however, were similar. On the other hand, patients in the GPI+ group had larger myocardial infarctions and hand worse pulmonary function. To address bias we performed a propensity score matching algorithm correcting for age, CPR duration, and SOFA score. Finally, the use of GPI remained to be an independent predictor of survival in a multivariate logistic regression analysis.

The biggest strength of his study is the liberal use of GPI therapy in cardiac arrest patients included in our registry. This liberal use has to be explained by the large time frame of the study dating back to 2003 and a liberal use of GPI at our center. With indication or GPI being limited to very specific diseases (with bad prognosis like no-reflow) by current guidelines, a register study performed nowadays might not be able to investigate the true potential of GPI in cardiac arrest ACS patients.

## Limitations

The decision for GPI therapy or other platelet inhibitors was not protocol based and left to the discretion of the operator. Therefore, an allocation bias has to be presumed. Data on platelet function is not available. The significant difference in the baseline characteristics in the two groups was addressed by propensity score matching and multivariate logistic regression analysis which both confirmed the results. Accordingly, we present retrospective data and results have to be considered hypothesis generating.

## Conclusion

This registry study of ACS patients after cardiac arrest and successful PCI suggest higher hospital survival rates in patients treated with GPI. Although more bleeding events were detected in the GPI+ group. GPI treatment seems to outweigh the bleeding complications. Still, these encouraging data have to be considered hypothesis generating.
